# Dielectric nanohole array metasurface for high-resolution near-field sensing and imaging

**DOI:** 10.1038/s41467-021-23357-9

**Published:** 2021-06-02

**Authors:** Donato Conteduca, Isabel Barth, Giampaolo Pitruzzello, Christopher P. Reardon, Emiliano R. Martins, Thomas F. Krauss

**Affiliations:** 1 Photonics Group, Department of Physics, University of York, York, UK; 2 São Carlos School of Engineering, Department of Electrical and Computer Engineering, University of São Paulo, São Carlos-SP, Brazil

**Keywords:** Biosensors, Optical sensors, Metamaterials, Imaging and sensing

## Abstract

Dielectric metasurfaces support resonances that are widely explored both for far-field wavefront shaping and for near-field sensing and imaging. Their design explores the interplay between localised and extended resonances, with a typical trade-off between Q-factor and light localisation; high Q-factors are desirable for refractive index sensing while localisation is desirable for imaging resolution. Here, we show that a dielectric metasurface consisting of a nanohole array in amorphous silicon provides a favourable trade-off between these requirements. We have designed and realised the metasurface to support two optical modes both with sharp Fano resonances that exhibit relatively high Q-factors and strong spatial confinement, thereby concurrently optimizing the device for both imaging and biochemical sensing. For the sensing application, we demonstrate a limit of detection (LOD) as low as 1 pg/ml for *Immunoglobulin G* (IgG); for resonant imaging, we demonstrate a spatial resolution below 1 µm and clearly resolve individual *E. coli* bacteria. The combined low LOD and high spatial resolution opens new opportunities for extending cellular studies into the realm of microbiology, e.g. for studying antimicrobial susceptibility.

## Abstract (web summary)

Dielectric metasurfaces have different Q-factor and light localisation requirements for sensing and imaging. Here, the authors present a dielectric metasurface, supporting two optical modes with sharp Fano resonances for high Q-factors and strong spatial confinement, allowing both sensing and imaging.

## Introduction

Metasurfaces consist of nanostructures that locally control the optical phase of a wavefront, a capability which is widely used for beamshaping^1,2^ in the far field. In many cases, the phase is controlled by optical resonances, which can be tuned by design^3^. These resonances should exhibit high *Q*-factors for maximum control and they need to be highly localised to achieve rapid phase changes across the surface^4^. The same requirements of high *Q*-factor and tight localisation apply to metasurfaces that are used in the near-field; a high *Q*-factor ensures that the resonant mode can sense refractive index changes very sensitively, and tight confinement ensures that the metasurface can act as a refractive index sensor with high spatial resolution, i.e. as an imaging sensor^5^. The *Q*-factor and localisation usually present a trade-off as one can typically only achieve one or the other.

This trade-off is easily understood by considering the paradigmatic guided-mode resonance (GMR) exhibited by a simple grating. The *Q*-factor of a GMR mode is determined by its scattering properties^6^ which in turn determine the penetration depth of the mode into the grating; a longer penetration depth yields a higher *Q*-factor^7^ and conversely, it reduces the spatial confinement. By exploiting resonances in unit cell, i.e. the meta-atom, this trade-off can be partially alleviated. For example, nanodisks^8^, elliptical dipoles^9^ or structures exploiting broken symmetry^10,11^ have shown promise. Nevertheless, the corresponding individual meta-atoms typically only support low-*Q* modes and the *Q*-factor of an ensemble increases as a function of array size^10^ akin to the original guided-mode resonance.

Since the first studies of protein sensing with plasmonic nanostructures^12–14^, significant improvements have been made by introducing the nanohole array configuration which enables the detection of proteins with sub-ng/ml concentration^15,16^. Further improvements are limited by the high losses of metals, however. Dielectric metasurfaces have therefore been introduced that support resonances with much higher *Q*-factors^17–19^. The highest experimental *Q*-factors so far have been achieved around *λ* = 1.55 µm^17^, but this wavelength range is not suitable for sensing and imaging, because sensing requires low-cost sources and detectors and because biological imaging is typically conducted in the visible range. Furthermore, *λ* = 1.55 µm is not suitable for high-resolution imaging, since spatial resolution scales with the operating wavelength. The *Q*-factor of *Q* = 450 at *λ* = 670 nm we report is therefore very competitive and it allows us to address two further aspects that have so far received little attention: (a) high resonance amplitude is essential for a high signal-to-noise measurement, yet much of the previous work has aimed to maximize the *Q*-factor alone; (b) the high sensitivity can also be most fruitfully applied to resonant imaging applications, which so far has been limited to mammalian cells due to their larger (5–10 µm) size^9,20,21^. A study aiming to concurrently optimize both the imaging and the sensing function is still missing.

**Fig. 1:**
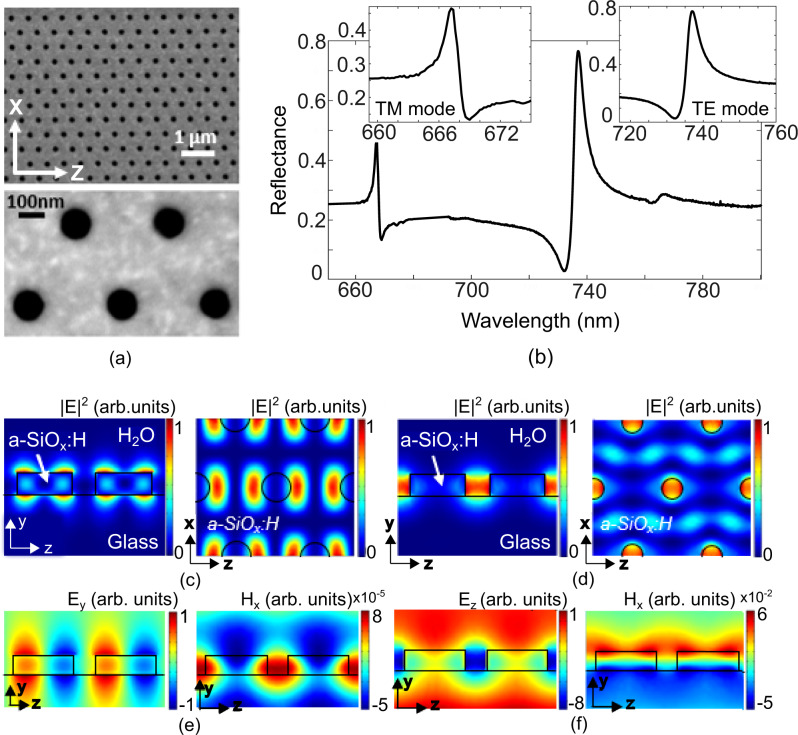
Configuration and results of the dielectric nanohole array. **a** SEM micrograph of the nanohole array realised in a-SiO_*x*_:H in a triangular lattice, showing overview and detail. **b** Experimental reflection spectrum of the nanohole array, highlighting the two main modes. **c** Cross-section (left) and top view (right) of the energy confinement for TM mode and **d** TE mode at resonance in the nanohole array. Cross-section of **e** the electric (left) and magnetic (right) field distribution for the TM mode and **f** the TE mode on resonance.

We now present a dielectric nanohole array in amorphous silicon that addresses these issues. Our structure supports two modes that combine a relatively high *Q*-factor with strong spatial confinement (Fig. 1), which addresses the trade-off between high spatial and spectral resolution and makes the device suitable for both imaging and sensing applications. Remarkably, this combination of *Q*-factor and localisation is inherent to the distributed mode of the periodic structure and not based on the introduction of defects or cavities. We discuss this interesting property and demonstrate its capability by showing high-sensitivity detection of immunoglobulin G (IgG) down to pg/ml concentrations, as well as quantitative refractive index imaging of individual *Escherichia coli* bacteria with a spatial resolution of better than 1 µm.

## Results and discussion

### Design and realization

The nanohole array (Fig. 1a) acts as a planar waveguide that supports 2D guided-mode resonances. It supports two optical modes with different profiles in the wavelength region of interest (Fig. 1b). The structure is realised in oxygen-enriched hydrogenated amorphous silicon (a-SiO_*x*_:H), which is transparent down to wavelengths as short as 650 nm (see ‘Methods’). The modes are excited with a normally incident collimated beam from an unpolarised halogen light source filtered through a monochromator; all spectra are normalised to a mirror. Both modes present a clear Fano lineshape of high *Q*-factor and high resonance amplitude (Supplementary Fig. 1).

We have designed the nanohole array in order to optimize the performance of both resonance modes concurrently, in particular aiming at improving the *Q*-factor, the sharpness of the Fano resonances and the resonance amplitude (see Supplementary Information 2 and 3).

We have calculated the field distribution of both resonant modes (Fig. 1), assuming an input field polarized along the *z*-axis, noticing that the reflection spectrum does not change by rotating the polarisation of the input field by 90°, i.e. to the *x*-axis. The excited modes can still be classified as TE or TM, since their effective index and field distribution closely resemble the TE and TM modes of the slab waveguide. Therefore, we define the TE or TM mode according to the mode distribution and not according to the input polarisation. The TM mode is resonant at *λ* = 667 nm with a *Q* = 450 (the *Q*-factor for a Fano resonance is defined as the spectral distance between the dip and the peak^22^) with a reflectance R_max_TM_ ~ 0.5. We refer to this mode as TM because its dominant E-field is oriented perpendicular to the surface. The second mode at *λ* = 737 nm exhibits a *Q* = 300 and has a higher resonance amplitude with a reflectance R_max_TE_ ~ 0.8. We refer to this as the TE mode because its dominant E-field is in the plane of the waveguide.

A remarkable advantage obtained with the dielectric nanohole array is a high value of SNR related to both modes. We define SNR_res_ = (*R*_max_−*R*_min_)/*σ*_spectrum_ with (*R*_max_−*R*_min_) the resonance amplitude and *σ*_spectrum_ is the standard deviation of the signal noise for both modes. Because of a high resonance amplitude and low noise values, we have verified SNR_TM _= 78 and SNR_TE_ = 160, with an evident improvement with respect to other plasmonic configurations and even compared to related dielectric metasurfaces (see Supplementary Information 4).

We have verified that both modes are suitable for imaging and sensing, however, their distinctive properties favour each mode for one of those applications. The field of the TM mode is extended in the lateral dimension, more akin to a typical GMR mode, while it is closely confined to the surface in the out-of-plane direction (Fig. 1c and e), which makes it more suitable for sensing. Conversely, the TE mode exhibits a high resonance amplitude and a very strong confinement to the holes, corresponding to an unusually strong localisation for a Bragg resonance, which makes it suitable for high-resolution imaging applications (Fig. 1d and f). We note that the existence of two different types of modes of distinct field distribution is a unique feature of a dielectric nanohole array, compared to a plasmonic nanohole array^23,24^ which only supports modes of a single polarisation^23^. We first consider the advantageous sensing properties of the TM mode before moving on to the imaging properties of the highly confined TE mode.

### Sensing

The commonly used figure of merit (FOM) for biosensing combines the sharpness of the resonance via its *Q*-factor with the sensitivity *S* of the resonance. The sensitivity is expressed as a wavelength change vs refractive index change Δ*λ*/Δ*n* in nm/RIU. So the FOM is *SQ*^25^. The sensitivity is commonly understood with respect to the bulk refractive index change, i.e. the response of the sensor to refractive index changes in the half-space above the sensor surface.

**Fig. 2:**
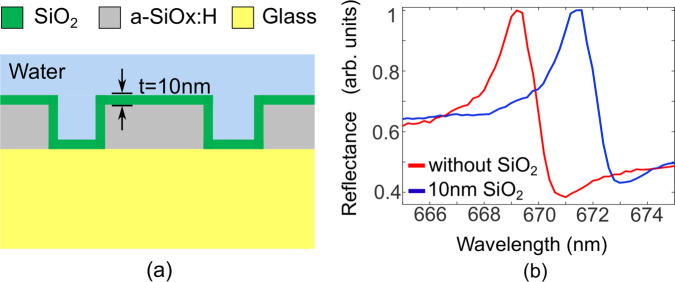
Measurement of the surface sensitivity. **a** Schematic of the cross-section of the nanoholes array with a SiO_2_ layer with a thickness *t* = 10 nm. **b** Experimental spectra of the bare nanoholes array (red curve) and with the SiO_2_ layer (blue curve) for TM mode.

Two-dimensional plasmonic nanohole arrays, for example, can achieve bulk sensitivities above 700 nm/RIU^23,26^ and dielectric nanohole arrays operating at 1550 nm can be even better with a demonstrated sensitivity of *S* ~ 800 nm/RIU^27^ and theoretically up to 4000 nm/RIU in the visible range^19^, while 1-D arrays typically achieve between 100 and 300 nm/RIU^28^. While the bulk sensitivity is easy to measure, it is not the most relevant parameter for a surface-affinity sensor; instead, the surface sensitivity should be used, which describes the response of the sensor to refractive index changes at the very surface of the sensor^25,29^ which is much more representative of surface-bound proteins or DNA. Since there is no agreed thickness for the surface sensitivity, we here chose a thickness of 10 nm and a layer of SiO_2_ (*n* = 1.45) to represent the thickness of a typical protein bound to a surface via an antibody^30^. By using amorphous silicon with a relatively high refractive index of *n* = 2.40 and exciting the TM mode, we confine the mode closely to the surface. We calculate the surface sensitivity as usual with *S* = Δ*λ*/Δ*n*, where in this case Δ*λ* represents the resonance shift between the bare nanohole array and the same structure with 10 nm SiO_2_ deposited on the structure, as described in Fig. 2a, while Δ*n* is the refractive index change between SiO_2_ (with the layer) and water (bare configuration). In both cases, water is assumed as the background medium. Using this method, we observe a surface sensitivity of 20 nm/RIU experimentally (Fig. 2b).

This value is comparable to that of plasmonic structures, which typically exhibit values of order 30 nm/RIU for a 10 nm layer^23^. While the difference between the two types of structures is rather large in terms of bulk sensitivity, it is surprisingly close in terms of the parameter that actually matters for surface biosensing, i.e. the surface sensitivity (Supplementary Information 4). In terms of the figure of merit *SQ*, we note that the *Q*-factor of the dielectric resonance is an order of magnitude (*Q* ~ 450, Fig. 1) higher than that of a plasmonic array (*Q* ~ 40^23^), resulting in a significantly higher *SQ* figure of merit when *S* is the surface sensitivity rather than the bulk sensitivity. Moreover, our nanohole array also compares favourably to related structures, such as the recently introduced structures based on bound states in the continuum (BIC) with *Q* = 90 and *S*_s_ ~ 40^9^. A higher FOM was also obtained with a dielectric nanohole array in ref. ^31^. However, as clearly shown in the foundational paper by Fan and White^29^, the SNR of the resonance curve additionally needs to be considered when assessing the performance of a biosensor, because it affords higher accuracy when tracking the resonance curve. We note that our nanohole array provides a significantly higher SNR_res_ for both modes compared to ref. ^31^, which contributes to the higher performance we describe.

For an overview of the different types of structures reported thus far, please refer to Supplementary Table 4.

We recognise that the *Q*-factor of any of these GMR-like structures is much lower than that of waveguide-based resonances such as microring resonators, but we note that waveguide-based resonances require excitation by end-fire coupling or with grating couplers, which require high precision coupling arrangements that are not compatible with the low-cost healthcare diagnostics approach that this work is aimed at.

In order to validate the advantageous properties of our approach, we conducted biological measurements using IgG as the target protein.

### Nanohole array for biosensing application

**Fig. 3:**
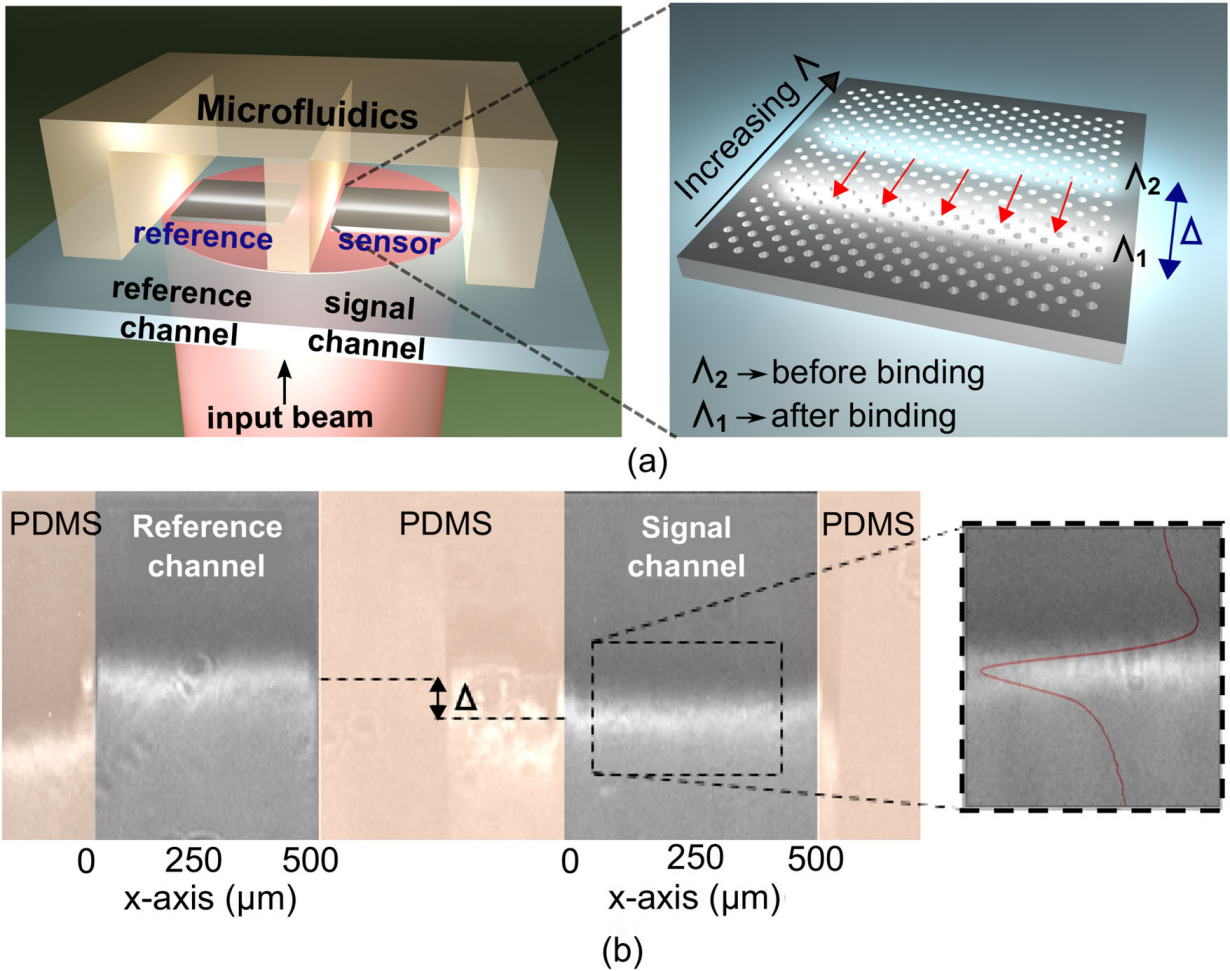
Nanohole array for biosensing application. **a** Schematic of the optical system with a nanohole array as sensor and a second microfluidic channel included for reference. The inset shows a schematic of the operation principle of a chirped nanohole array with a shift of the resonance position due to a change of the effective index for protein binding. **b** Camera image of 2 chirped nanohole arrays in the reference and signal channels for an input wavelength of *λ* = 667 nm. The shift Δ of the resonance position occurs after IgG binding. The inset shows the Fano fit (red curve) of the resonance curve.

For the biological measurements, we adopted the chirped configuration^32,33^ to the nanohole array for ease of read-out. This configuration is obtained by tapering the nanohole period from Λ = 470 nm to Λ = 490 nm over a distance of 500 µm. Accordingly, for single wavelength illumination, the resonance will appear as a bright line at the output of the chirped array, spatially located where the period multiplied by the effective index matches the wavelength and is able to excite a resonance (Fig. 3). Any binding of the target biomarker to the sensor surface then causes a shift of the position of the line due to the change in the effective index, thereby translating spectral into spatial information (Fig. 3a). This information is then easily read-out by a CMOS camera. In order to make the system immune to temperature variations of the environment, we include a second microfluidic channel as a reference (Fig. 3b). This reference channel is not functionalised and is exposed to a buffer solution. The differential measurement between the signal and reference channels provides the resonance shift caused by the antigen binding to its specific antibody.

The bulk sensitivity of the sensor is 140 nm/RIU (we here include the bulk sensitivity for ease of comparison with literature values) corresponding to a sensitivity for the chirped array of 3960 µm/RIU, defined as the spatial shift of the resonance position per unit change of the bulk refractive index.

The high resonance amplitude for both modes represents a further advantage of the dielectric nanohole array compared to its plasmonic counterpart, which has a much lower resonance amplitude with a typical transmission of *T* < 0.2. The high resonance amplitude is comparable to that observed with 1-D GMRs^28,34^, but we note that we achieve a significantly higher *Q*-factor (*Q* ≈ 450) compared to *Q* ≈ 150–250 for a 1-D GMR. The high *Q*-factor, together with the high resonance amplitude, produces a sharp and bright resonance line as shown in Fig. 3. From the binding assay, we extract a noise limit of 3*σ* = 0.183 µm over 30 min, corresponding to approximately the time it takes to reach saturation when detecting low protein concentrations.

Together with the sensitivity of 3960 µm/RIU mentioned above, this noise limit translates into a limit of detection of 4.6 × 10^−5^ RIU (Supplementary Information 5).

For the surface functionalisation, we use a spacer layer of *SM(PEG)6* (succinimidyl-[(N-maleimidopropionamido)-hexaethyleneglycol] ester) between the sensor surface and the antibodies to decrease non-specific binding^35^ (see ‘Methods’).

**Fig. 4:**
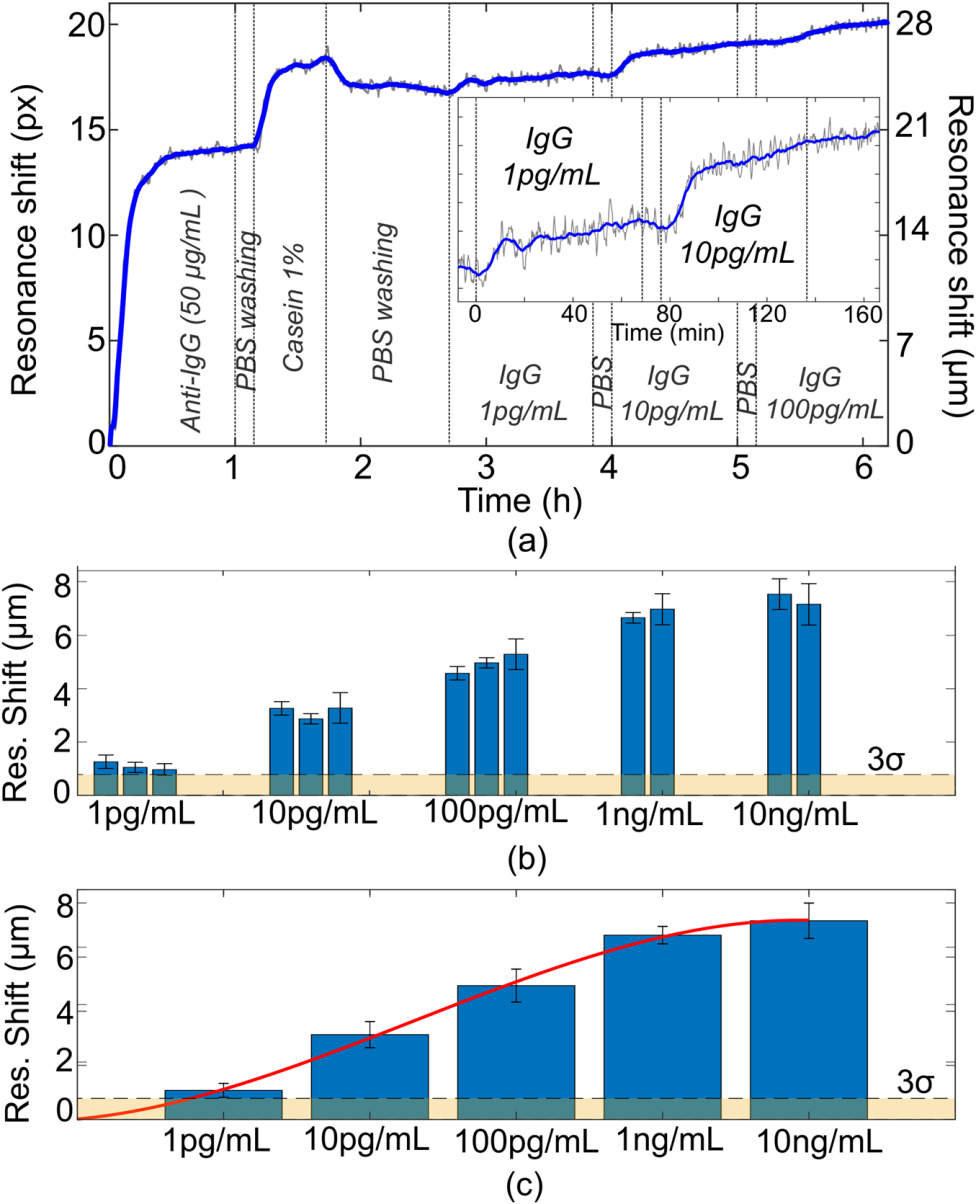
IgG detection with the nanohole array. **a** Binding assay for IgG measurements with PEGylated functionalized surface of nanoholes array with TM mode. **b** Histograms of the resonance shift for different IgG concentrations with multiple measurements for each case. The error bars represent the deviation standard from the average value for each measurement. **c** Average resonance shift for different IgG concentrations and fitting of the binding assay in red curve. The error bars represent the maximum deviation from the average value of multiple measurements for each IgG concentration. The value 3*σ* is highlighted by the dashed line both in **b** and **c**.

We use IgG, a generic marker for the human immune response, to quantify the protein sensing performance of the nanohole array. The binding assay (Fig. 4a) includes the surface functionalization with anti-IgG antibodies immobilized on the *PEG* layer, followed by casein as an additional blocker against non-specific binding to optimize the sensor specificity, as previously verified in ref. ^35^ (see ‘Methods’).

Following functionalisation, we added different IgG concentrations (1, 10, 100 pg/mL, all in phosphate buffer saline (PBS)) to the channel. In particular, for a concentration of 1 pg/mL, we observe a shift of 1.26 µm (=0.9 pixels), corresponding to a wavelength shift of about Δ*λ* ~ 30 pm, while the noise level in this specific measurement is 3*σ* = 0.78 µm (=0.56 pixels). We have taken multiple measurements for each IgG concentration and we note a resonance shift for 1 pg/mL higher than the 3*σ* value, confirming a limit of detection better than 1 pg/mL (Fig. 4b). The sensor dynamic range, corresponding to the range from the lowest to the highest measurable analyte concentration in the bioassay measurement, is from 1 pg/mL to 10 ng/mL (Fig. 4c). This high performance is in part due to the use of the *SM(PEG)6* spacer layer which we first introduced to GMR-based sensing in ref. ^35^, where we also showed very low non-specific binding and high-sensitivity detection in human urine. The control experiments to verify the specificity of the biosensors are reported in Supplementary Information 6. The time required to reach the saturation for the antigen binding is <20 min and it depends on the efficiency of the surface functonalisation and, in particular, on the binding efficiency between the antibodies and the antigen, and not directly on the metasurface performance itself.

Remarkably, the demonstrated performance with LOD < 1 pg/mL is comparable to or better than the laboratory standard, i.e. fluorescence-based enzyme-linked immunosorbent assay (ELISA), which typically achieves sensitivities of the 3–5 pg/mL^36^, yet our label-free and very simple approach is more suited for point-of-care applications. It also represents an improvement by over two orders of magnitude compared to plasmonic nanohole array^16^ and is even better than a sandwich assay using metal nanoparticles based on a similar structure^37,38^ or based on other dielectric metasurfaces^9,39^. We explain this improvement of performance with the high surface sensitivity, the high values of *Q*-factor and SNR, together with a sharp Fano resonance, which facilitates easy tracking of the resonance.

We have recently also demonstrated an interferometric approach based on guided-mode resonances^40^ where we have shown the detection of 1 pg/ml of procalcitonin (PCT) with very high SNR. We note that the main advantage of the nanohole array configuration shown here is its ability to obtain a similar sensing performance together with the imaging capability. It is interesting to note that the TE mode also exhibits high sensitivity, i.e. its detection limit for *IgG* is comparable to that of the TM mode (Supplementary Information 7), which is relevant for the following imaging section. We note, however, that the TM mode performs better in terms of reproducibility and signal-to-noise, which we attribute to the mode distribution being more suitable for detecting surface-bound molecules.

### Single bacteria detection with resonant hyperspectral imaging

We now address the spatial confinement and refer to the TE mode (Fig. 1d). We note that the high resonance amplitude obtained with this mode should translate into high contrast imaging and thus improves the resolution of imaging^41^. Furthermore, the fact that the optical field is strongly confined in the holes and less distributed on the surface improves the spatial resolution, which suggests the suitability of the TE mode for imaging. In order to verify this hypothesis, we use hyperspectral imaging applied to a standard configuration of a dielectric nanohole array with fixed period providing the same resonance condition over the entire sensor area, whereby the wavelength of the light source is scanned and images are taken at every wavelength step with a CMOS camera (see ‘Methods’). The peak wavelength of the resonance is subsequently extracted for each pixel as the wavelength that maximizes reflectance^9,42^. The spatial resolution of this method, for a dielectric 1-D GMR is typically of the order 2–6 µm^34,43^, which is usually limited by the penetration depth of the GMR into the grating, so scales with *Q*-factor as discussed above. Therefore, a worse spatial resolution for the higher *Q*-factor of our nanohole array would be expected, yet surprisingly, we observe the opposite. As an aside, we note that a higher resolution as low as 0.5 µm has already been quoted with dielectric configurations^42^. However, such values refer to the identification of point sources, not the separation of two features, which instead is the commonly accepted method underpinning the Rayleigh criterion.

In order to test the resolution of the nanohole array, we first (as in ref. ^34^) deposit a high-resolution pattern and test for the separation between closely spaced features using hyperspectral imaging. We are able to observe an imaging resolution of better than 1 µm, which confirms the strong spatial resolution of the TE mode (Supplementary Information 8).

We have also evaluated the imaging performance of the TM mode and obtained a spatial resolution of about 3 µm. As expected, the lower spatial resolution is mainly due to the lower SNR and the weaker localisation of the mode (Supplementary Information 9).

**Fig. 5:**
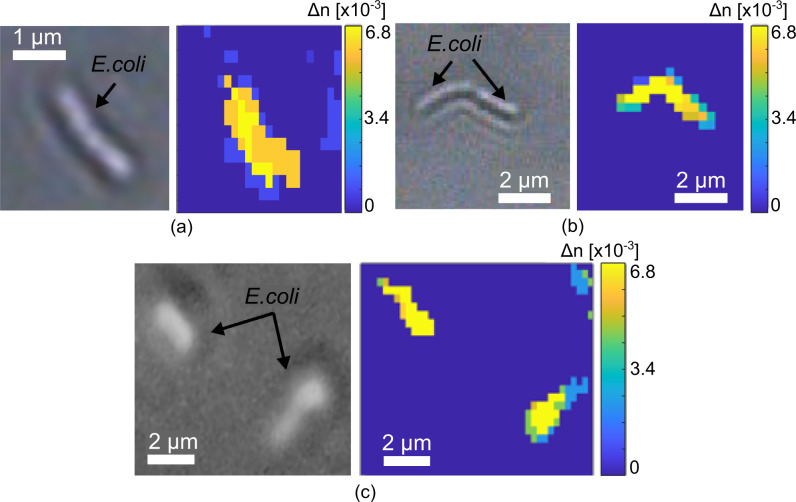
Imaging of individual *E. coli* bacteria. **a**–**c** Microscope images (left) and hyperspectral imaging (right) of individual *E. coli* bacteria.

Encouraged by these results, we turn to applying this high resolution to biological studies. Figure 5 shows that we can clearly resolve the shape and orientation of individual *Escherichia coli* bacteria (typical size of about 2 × 1 µm) with high accuracy.

We demonstrate that our technique provides quantitative information with high spatial resolution by imaging and spatially localizing individual bacteria (Fig. 5 and Supplementary Information 10), enabling the detection of the refractive index changes caused by the presence of bacteria. We use the refractive index calibration determined for sensing (Figs. S5, S7) to achieve quantification. We note the ability of the structure to provide imaging information with a refractive index resolution of 4.6 × 10^−5^ RIU, which is not reachable with conventional imaging techniques. These results demonstrate that the many attractive features of resonant hyperspectral imaging for studies involving, e.g. adhesion, secretion and cell viability previously demonstrated with mammalian cells^20,21,44^ can now also be applied to bacteria. Individual bacteria have already been studied with traditional techniques based on the use of fluorescent dyes^45^. Furthermore, photonic nanostructures have been also used for nanoscopy on-chip for imaging the membrane/cytoskeleton interactions in single cells^46^, providing a resolution better than 100 nm, beyond the diffraction limit, with a field of view at millimetre scale, but only combined with fluorescence. However, the labelling process associated with these techniques complicates the procedure and may distort the result. In contrast, the nanohole array is based on a completely label-free approach and, in particular, because the structure is defect-free, the sensing area can be very large (up to few mm^2^, only limited by the field of view of the camera). This allows for the real-time monitoring of bacterial growth in a large area (see Supplementary Information 11) while preserving a sufficiently high resolution to image individual bacteria. This capability is particularly important, e.g. for studying the formation of biofilms and for testing antimicrobial susceptibility, a major problem in the quest to control antimicrobial resistance (AMR).

To summarize, we have introduced a dielectric metasurface based on a nanohole array realised in amorphous silicon. The key feature of the array is that it supports two distinct modes in the region of interest, both of which exhibit sharp Fano resonances and distinct field distributions that offer high performance for sensing (TM mode) and for imaging (TE mode). We have used the TM mode with its high *Q*-factor and high surface sensitivity to demonstrate sensing of IgG proteins with a limit of detection of better than 1 pg/ml, while the high resonance amplitude and strong confinement of the TE mode have been used to demonstrate hyperspectral imaging with a spatial resolution of better than 1 µm and resonantly resolve single bacteria.

Being able to maximize sensitivity and spatial resolution with the same structure by simply selecting the most appropriate resonant mode, because of the different types of modes being supported, is unique to this system, as e.g. compared to plasmonic nanohole arrays or other dielectric metasurfaces demonstrated thus far. We believe that the dielectric nanohole array geometry with the ability to reach high-resolution refractive index imaging will open new opportunities for sensing and imaging with a label-free approach, especially for the study of bacteria in combination with their surrounding specific molecule distribution which were previously challenging to resolve with comparable structures.

## Methods

### Nanohole array fabrication

The nanohole array is realized in oxygen-enriched hydrogenated amorphous silicon (a-SiO_*x*_:H). Pulsed DC magnetron sputtering is used for the deposition of a 110-nm thick film on a 500 µm borofloat glass substrate in the presence of hydrogen and residual oxygen in the chamber. The sample is cleaned in a piranha solution (1:3 hydrogen peroxide:sulfuric acid) for 10 min and rinsed in deionized water, acetone and isopropanol. The structure is defined by electron beam lithography followed by reactive ion etching. The resist used for the e-beam exposure is *ARP-13* (Allresist GmbH) spincoated at 5000 rpm for 60 s and soft baked on a hot plate at 180 °C for 5 min. A 60-nm layer of *AR-PC* 5090 (Allresist GmbH) is spincoated at 2000 rpm for 60 s and baked at 90 °C for 2 min for charge dissipation during the e-beam exposure. For the e-beam exposure (Voyager; Raith GmbH) we use a dose of 190 µC/cm^2^. The sample is then washed in deionized water to remove the charge dissipation layer and the resist is then developed with xylene for 150 s, rinsed with isopropanol and dried with nitrogen. The pattern of the nanoholes is then transferred into the a-SiO_*x*_:H layer by reactive ion etching with a gas mixture of CHF3:SF6 = 14.5 sccm:12.5 sccm for 1 min with a voltage *V* = 188 V and a chamber pressure of 0.4 mbar. Finally, the remaining resist is removed by sonicating the structure at 50 °C in 1165 solvent (MicroChem) for 5 min, followed by a rinse of 5 min in acetone and isopropanol and a final drying step with nitrogen.

### Fabrication of the microfluidic channels

The microfluidic channels are made in polydimethylsiloxane (PDMS) elastomer (Dow Corning). The mould for the channels is realized with SU-8 2050 resist (Microchem) spincoated on a silicon substrate at 1000 rpm for 60 s and soft baked on a hot plate at 65 °C for 5 min and then 95 °C for 30 min, with a final thickness of 170 µm. We use direct UV laser writing (Kloé Dilase 650) to define the pattern of the mould for the channels in the resist, followed by a post-baking at 65 °C for 5 min and then 95 °C for 12 min. The resist is developed for 15 min in EC solvent and rinsed in IPA. Finally, the mould is hard baked in the oven at 180 °C overnight. For the channels in PDMS, we use a ratio 7:1 elastomer:curing agent. The mixture is desiccated for 30 min and then poured on the SU-8 mold and finally in the desiccator for further 30 min. The PDMS is cured overnight in the oven at 60 °C and then carefully peeled off from the mold. After curing, holes are punched through the PDMS for the inlet and outlet sections, and both the sensor chip and PDMS channels are left for 100 s in oxygen plasma and then bonded irreversibly with a final curing step in the oven at 60 °C for 3 h.

After the integration of the microfluidics on the sensor chip, tubes (Tygon) are inserted in inlets and outlets sections and directly connected to a syringe-pump (SPLG210) to enable the liquid flow in the channels.

### Optical setup for the characterization of the nanohole array and hyperspectral imaging

The optical setup to characterize the nanoholes array includes a Halogen source (ASBN-W, Spectral Products). The input beam is sent through a monochromator (Digitkrom, Spectral Products) in order to select a single wavelength with a spectral resolution of 0.3 nm. The beam is then focused by a Köhler lens in the back focal plane of an ×4 objective lens (Olympus PLN4X with NA = 0.1) to obtain a collimated beam used to illuminate the photonic structure. The beam reflected from the sample is then redirected by a beam splitter to a camera (Quantalux sCMOS, THORLABS) where each pixel corresponds to 1.4 µm. Labview® software is used to control the monochromator to sweep the wavelength over a range from *λ* = 600 nm to *λ* = 800 nm with a step of 0.3 nm and the corresponding brightfield images are saved for each wavelength to allow reconstruction of the reflection spectrum for each pixel. The final spectrum is obtained by normalizing the signal from the GMR structure to a reference signal that has been measured in parallel. The same setup is used for biological measurements, except for a fixed position of the monochromator to illuminate the structure at a single wavelength. The camera images are processed to fit the optical intensity traces to a Fano profile in order to accurately define the position of the reflection peak with sub-pixel resolution. The resonance wavelength is tracked every 15 s.

The same setup is also used for the hyperspectral imaging of bacteria but with an ×20 objective lens (Olympus PLN20X with NA = 0.4) to increase the magnification for a better spatial resolution. Each pixel in this condition corresponds to 0.45 µm (Fig. 5b and Fig. 5c) and 0.2 µm (Fig. 5a) with an extra lens before the CMOS camera for a further magnification. A time lapse of 4 min is used to image the bacteria growth over a total time of 4 h.

### Specific surface functionalization for immunoglobulin-G detection

The functionalization protocol of the photonic biosensor needs to provide high specificity to the target molecule, while preventing the binding of non-targeted molecules to avoid unspecific binding. In order to improve the binding efficiency, we use a *SM(PEG)6* as spacer between the surface of the nanoholes array and the antibodies. This spacer ensures high uniformity for the bonding of antibodies covering a large area of the sensor, with a reduction of steric hindrance and aggregation. The functionalization protocol includes a first cleaning process of the sensor with a piranha solution (H_2_SO_4_:H_2_O_2_ = 3:1) for 10 min followed by UV-Ozone surface cleaning treatment for 30 min. The piranha process introduces hydroxyl groups (OH) on the surface and the surface is then covered by sulfhydryl groups by the salinization with (3-Mercaptopropyl)trimethoxysilane (MPTS) for 6 h. The spacer monolayer *SM(PEG)6* is then formed during an overnight process with a 1 mM PEG in DMSO solution. The *SM(PEG)6* not binding to MPTS is then removed by a washing process in DMSO. At this step, the antibodies anti-IgG (with a concentration of 50 µg/mL in PBS) are introduced in the signal channel with a continuous flow at 20 µL/min for 60 min. A PBS washing step (5 min) follows to remove any antibody not bound to the *PEG* spacer. The casein (1% in PBS solution) is then added into the channel for 30 min to minimize unspecific binding, followed by a washing step in PBS for 60 min. Three different concentrations of IgG are released into the channel at room temperature (*T* ~ 20 °C). Each concentration is introduced with a flow at 20 µL/min for 60 min, followed by a washing step in PBS for 5 min.

### Bacteria culture and sensor surface functionalization for bacteria immobilization

The bacteria we use are *Gram*-negative *Escherichia coli TG1*. The bacteria are grown overnight in lysogeny broth (LB, 10 g/L NaCl, 10 g/L tryptone and 5 g/L yeast extract) at 37 °C. The resulting solution is then diluted in LB to reach a concentration of ~10^6^ CFU/ml. We use a spectrophotometer to obtain an optical density OD = 0.01 corresponding to the desired initial concentration. A droplet of 50 µl is then applied to the sensor surface with a pipette and covered by a glass coverslip to avoid evaporation during the 4 h duration of the experiment. The functionalization protocol is the same as that used to functionalize the sensor surface for the IgG detection with a first step by covering the surface with MPTS for 6 h to create the sulfhydryl groups, where *SM(PEG)6* binds after an incubation in *DMSO* overnight. After washing, a drop of 60 µL of anti-*E. coli* antibodies (*E. coli* antibody (1011), Santa Cruz Biotecnology) is released on the sensor for 1 h to allow the bacteria immobilization on the surface. Finally, the sensor is washed in PBS and bacteria are incubated for *t* = 6 h on the sensor at room temperature.

## Supplementary information

Supplementary Information

## Data Availability

The data that support the findings of this study are available from the corresponding author upon reasonable request.

## Code availability

All codes produced during this research are available from the corresponding author at reasonable request.
