# Supplementary material for: Dielectric nanohole array metasurface for high-resolution near-field sensing and imaging
Source: Nat Commun. 2021 Jun 2;12:3293. doi: 10.1038/s41467-021-23357-9 (PMC8172834; doi:10.1038/s41467-021-23357-9)
Supplement: Supplementary file 1 — Supplementary Information [file 41467_2021_23357_MOESM1_ESM.pdf]

Supplementary Information

Dielectric nanohole array metasurface for high-resolution near-field sensing and imaging

Donato Conteduca[1*], Isabel Barth[1], Giampaolo Pitruzzello[1], Christopher P. Reardon[1], Emiliano R. Martins[2], Thomas F. Krauss[1]

1. Photonics Group, Department of Physics, University of York, Heslington, York YO10 5DD, UK
2. São Carlos School of Engineering, Department of Electrical and Computer Engineering, University of São Paulo, São Carlos-SP, Brazil

**e-mail: donato.conteduca@york.ac.uk*

Supplementary Notes

Supplementary Note 1: Design and experimental results of the nanohole array

The nanohole array is designed as a triangular lattice (α = 60^0) of period Λ_x = 480 nm realized in a-SiOx:H (n = 2.40 and k = 5x10^{-4} @ λ = 700 nm) with a thickness *t* = 110 nm on a glass substrate (Supplementary Figure 1). The optical properties of the material are determined with an ellipsometer. Each hole has a diameter *D* = 120 nm. The COMSOL® Multiphysics finite element method is used to simulate the optical response of the device. The unit cell is shown in Supplementary Figure 1a and periodic boundary conditions are used. A plane wave is launched in normal direction to the plane of the array to replicate the experimental conditions. Perfectly matched layers (PMLs) are used at the top and bottom of the computational domain to prevent spurious reflections. The resulting reflection spectrum including both the TM and the TE modes is shown in Supplementary Figure 1b. The numerical results show a Q-factor = 550 for the TM mode with a resonance wavelength at λ = 665 nm and Q = 400 for the TE mode with a reflection peak at λ = 735 nm. The experimental spectrum of Fig. 1b (main manuscript) is superimposed to highlight the excellent agreement with the simulation.

Both modes provide a sharp Fano resonance confirmed by the low values of the damping parameter and resonance asymmetry [18], that we associate with the transparency of the dielectric material, obtaining a relevant improvement compared to Fano resonances in plasmonic devices. Furthermore, an evident increase of both parameters is observed when increasing the optical losses of the material, as expected, with a consequent fast decrease of Q-factor and smaller resonance amplitude. We also note that the experimental trace for the TM mode at 665 nm exhibits a relatively lower reflectance amplitude than

the TE mode, which we associate with its higher sensitivity to surface roughness, which is not included in the model.

Supplementary Note 2: Optical losses and angular dependence

We measured a loss value of k ~ $5x10^{-4}$ of the a-SiOx:H material used in the experiments and the experimental and numerical spectrum are shown in Supplementary Figure 1. We have also estimated the effect of optical losses on the performance of the metasurface, in particular on the Q-factor and reflectivity (Supplementary Figure 2a). Numerical results show that both modes present high values of reflectivity (R > 0.9) together with relatively high Q-factor with k $<10^{-3}$ (Supplementary Figure 1b). In this condition, there is almost a negligible change of Q-factor and only the reflectance slightly reduces. A different composition of the material with a decrease of oxygen and hydrogen content would allow to increase the refractive index but at the expense of a relevant increase of the optical losses (k~10^{-2}). Therefore, the material composition used to realise the metasurface represents the best compromise to minimise the optical losses with the maximum value of refractive index that we can achieve.

We also calculated the angular dependence of the performance of the metasurface (Supplementary Figure 2b). The numerical results show that there is a slight shift of the resonance curves as a function of input angle but that the two dominant modes we refer to in the paper are largely preserved; also, a change in input angle of 2° is well above the tolerance of our setup, so we can safely conclude that the angular dependence does not present any issues with our interpretation. More interestingly, we note that new modes appear for angular incidence, which are symmetry-forbidden at normal incidence, but studying these in detail is well beyond the scope of the paper.

Supplementary Note 3: Design optimization of the dielectric nanohole array

A rigorous design of the dielectric nanohole array has been carried out to optimize the performance of both resonant modes concurrently. We have investigated a parametric analysis on the period, hole radius and slab thickness to quantify the Q-factor and the resonance amplitude for the TE and TM mode.

The optimized structure should provide a high Q-factor and wide resonance amplitude for the TM mode used for sensing application, while extremely high resonance amplitude and strongly localized energy confinement are required for the TE mode for imaging applications. A strong Fano-shaped resonance improves the performance of both modes for the target applications.

We have chosen a triangular lattice compared to a square lattice because it provides slightly higher values of Q-factor and resonance amplitude for both TM and TE mode. We have chosen Λ = 480nm and R = 60nm as the optimized configuration, because it represents the best compromise between high resonance amplitude and high Q-factor. In particular, the Q-factor increases with a larger lattice period Λ and a smaller hole radius for both resonant modes (Supplementary Figure 3a and 3c). However, as the hole radius decreases, the resonance amplitude decreases as well, which is counterproductive (Supplementary Figure 3b and 3d). We note that other work, driven by the SQ figure of merit alone, tends to ignore this trade-off. We have therefore chosen a radius of 60 nm as a good compromise, which is also technologically feasible with our lithography and dry etching process. The choice of the thickness t = 110 nm is justified by the fact that a lower thickness provides a higher Q-factor but at the expense of a fast decrease of reflectance (Supplementary Figure 3e*)*, so it presents a similar trade-off as the hole radius.

Supplementary Note 4: Comparison of the performance of dielectric nanoholes with the state-of-the-art of label-free measurements with comparable structures

In Supplementary Table 4, we compare the performance of the dielectric nanohole array with the State-of-the-Art of different configurations used for sensing in the visible and in the near-infrared wavelength range. We compare the resonance amplitude (R_{max}-R_{min}) which leads to the signal-to-noise ratio of the resonance ($SNR_{res}=(R_{max}-R_{min})/\sigma$), the figure of merit FOM = SQ, where Q is the Q-factor and S the surface sensitivity of the sensor and the LOD, as the minimum detectable concentration for biosensing. While the dielectric nanohole array offers a lower surface sensitivity than some of the other configurations, the Q-factor is typically higher, which yields a higher FOM overall. We also note that the 1D-GMR of [28] is comparable to our results in terms of the FOM, but it operates using a very

distributed mode, which consequently has poor spatial resolution. A higher value of SQ has been obtained in [31] with a dielectric nanohole array exploiting the BIC modes. However, we note that our nanohole array provides a significant enhancement of the SNR_{res} for both modes, which plays an important role in the optimization of the imaging and sensing resolution, as described in detail below. In addition, the functionalisation protocol adds further variability, which highlights that the FOM quoted in many papers is only part of the story and that biosensors should mainly be compared on their LOD for biological measurements. Since spatial resolution is not mentioned in most papers, it has not been included here, but we note that the dielectric nanohole array offers a very favourable combination of sensing and imaging performance.

Supplementary Note 5: Bulk sensitivity and limit of detection of the chirped nanohole array

We determine the bulk sensitivity of the chirped nanohole array by changing the refractive index of the solution in the channels; different refractive indices are obtained via different concentrations of glucose in DI water (Supplementary Figure 5a). We observe a linear shift of the resonance position as a function of refractive index in the range from $\Delta n = 0.001$ to $\Delta n = 0.023$ with a minimum shift of 5.73 μm for $\Delta n = 0.001$ compared to DI water ($n_{H20} = 1.3329$). We use these results to obtain the sensitivity of 3960 μm/RIU (Supplementary Figure 5b) stated in the main manuscript. In order to translate this figure to the more commonly used sensitivity in nm/RIU, we also measured the bulk sensitivity of the array by compensating for the shift due to refractive index with a change in illumination wavelength. For the maximum refractive index change of $\Delta n=0.023$, we require a wavelength change of $\Delta\lambda = 3.37$ nm, corresponding to a bulk sensitivity S = 140 nm/RIU. This value is in good agreement with the sensitivity obtained from a non-chirped array of the same period. In order to determine the noise limit, we measure for 30 minutes in DI water and obtain a value $3\sigma = 0.183$ μm (3 times the standard deviation) (Figure Supplementary Figure 5c). Together with the 3960 μm/RIU, this corresponds to a limit of detection of LOD = $4.6\text{x}10^{-5}$ RIU.

Supplementary Note 6: Biosensor specificity

We have conducted a control experiment to verify the specificity of our biosensor. We have assumed the same chirped configuration of the dielectric nanoholes array in both the signal and the reference channel. In this control experiment we flow IgG in both channels, which are functionalised respectively with the specific antibody anti-IgG and unspecific antibody anti-CRP (Supplementary Figure 6). We observe an evident resonance shift with 20 μg/mL IgG concentration in the signal channel (blue curve) functionalised with the specific antibody anti-IgG, while a negligible shift lower than the noise signal is observed with the same IgG concentration in the reference channel (red curve) functionalised with anti-CRP. The sensor behaviour confirms high specificity for protein detection.

Supplementary Note 7: Comparison of sensitivity of the TE and TM modes for biosensing

We have also studied the optical response of both optical modes for biosensing applications. A comparison of the binding curves in the presence of 10 pg/mL of IgG is shown in Supplementary Figure 7. The resonance shift observed with this concentration is almost the same for both modes. However, the noise for the TM mode is much lower ($3\sigma = 0.78$ μm), i.e. approximately 2 times lower than for the TE mode in the same measurement. We believe that this difference can be explained with the higher Q-factor of the TM mode which affords more accurate tracking of the resonance. Moreover, the TM mode distribution is much more suitable for surface sensing than that of the TE mode, which is mainly confined to the holes (Figure 1c, d in the main manuscript). The field of the TM mode offers a larger area for interaction with surface-bound molecules, which makes for a more reliable measurement. In contrast, the TE mode mainly interacts with molecules having diffused into the holes, which tends to be more erratic; as a result, we have observed less consistency with TE mode measurements on multiple repeats of the experiment. Nevertheless, the fact that the TE mode exhibits similar performance as the TM mode makes it very suitable for measurements that require a combination of strong localisation and high sensitivity, as e.g. for the studies of bacteria.

Supplementary Note 8: Resolution of hyperspectral imaging

We evaluate the spatial resolution of the nanohole array by lithographically defining blocks of different refractive index on the surface (Supplementary Figure 8a). We then use hyperspectral imaging to resolve the size and the position of the blocks by analysing the spectral information exploiting the TE mode.

As explained in the Methods, multiple images are obtained at different wavelengths in order to construct a hyperspectral cube (Supplementary Figure 8b and 8c). The higher refractive index of the blocks causes a resonance shift in the block region. In order to determine the spatial resolution of this resonance shift, we made a symmetric structure consisting of square blocks of SiO_2 with a width of 5 µm and a gap size of 0.9 µm, 1.4µm, 2.9µm (Supplementary Figure 8a). The experimental results confirm that a gap size of 0.9µm can still be resolved in both directions, which shows that a spatial resolution less than 1µm is obtained. (Figure S8d and Figure S8e). This result compares favourably to previous results in 1-D GMRs, which exhibit a spatial resolution of 2-6µm depending on orientation [Refs. 20, 21, 34 in the manuscript].

Supplementary Note 9: Study of the spatial resolution for both resonant modes with hyperspectral imaging

We have evaluated the spatial resolution with the nanohole array for both resonant modes (Supplementary Figure 9). The high resonance amplitude obtained with the TE mode provides a strong image contrast with the blocks on resonance (Supplementary Figure 9f), which together with its stronger confinement, enables resolving the blocks and the gap between them with higher accuracy than the TM mode. While the spatial resolution for the TE mode is around 1 µm in both directions (Supplementary Figure 9a and 9b), the TM mode does not achieve such high resolution, due its more distributed confinement and its lower resonance amplitude (Supplementary Figure 9c and 9d). Correspondingly, for the TM mode, we note a spatial resolution of approximately 3 µm (Supplementary Figure 9e).

Supplementary Note 10: Optical response of single bacteria

Supplementary Figure 10 shows a comparison between the resonance behaviour for a bacterium binding to the surface and the background without bacteria. We observe a clear shift of the resonance ($\Delta\lambda = 3.4$ nm) for the bacterium on the sensor surface, which allows us to resolve the size and position of the bacterium. The observed shift is remarkably higher than the threshold value of 0.5 nm, which we have assumed as a baseline in the hyperspectral analysis (in order to consider possible inhomogeneity in the surface chemistry and experimental errors), and which provides a clear and high-resolution hyperspectral map for the detection of individual bacteria.

Supplementary Note 11: Hyperspectral imaging for the monitoring of the bacteria growth on the nanohole array

The nanohole array with a resolution down to 1μm and a field of view up to few mm^2 is suitable for the monitoring of the growth of bacteria. Supplementary Figure 11 shows the time evolution of the hyperspectral map for studying bacterial growth in a region of interest of 50 μm x 50 μm of the nanohole array. The presence of a first colony (*A*) is evident in the region of interest after 1 h and further covering occurs over time. A similar behaviour is observed for the growth of different colonies (*B*) and (*C*) in different regions and time frames with a faster growth rate after 4h due to the stronger interaction between different colonies. A control experiment with the nanohole array in LB medium without bacteria for the same time scale demonstrates negligible change in the hyperspectral map, confirming that the resonance change is actually due to the presence of bacteria on the sensor.

Supplementary Figures

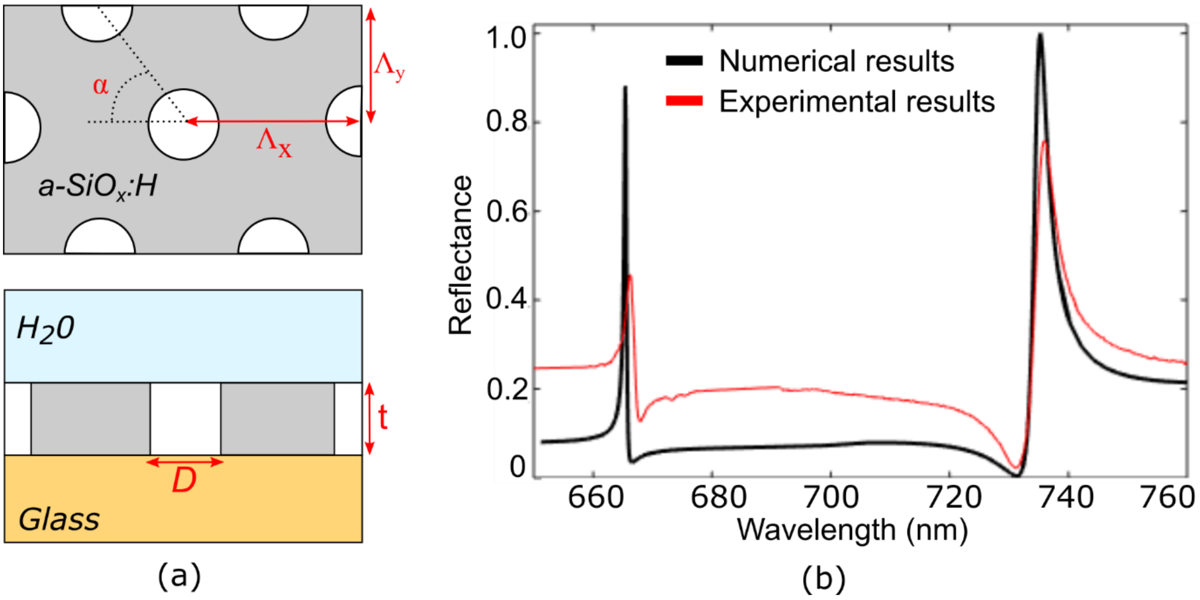

Supplementary Figure 1. (a) Top view (top) and cross-section (bottom) of the nanohole array with a triangular lattice and (b) reflection spectrum calculated using COMSOL® Multiphysics (black line) and experimental results (red curve).

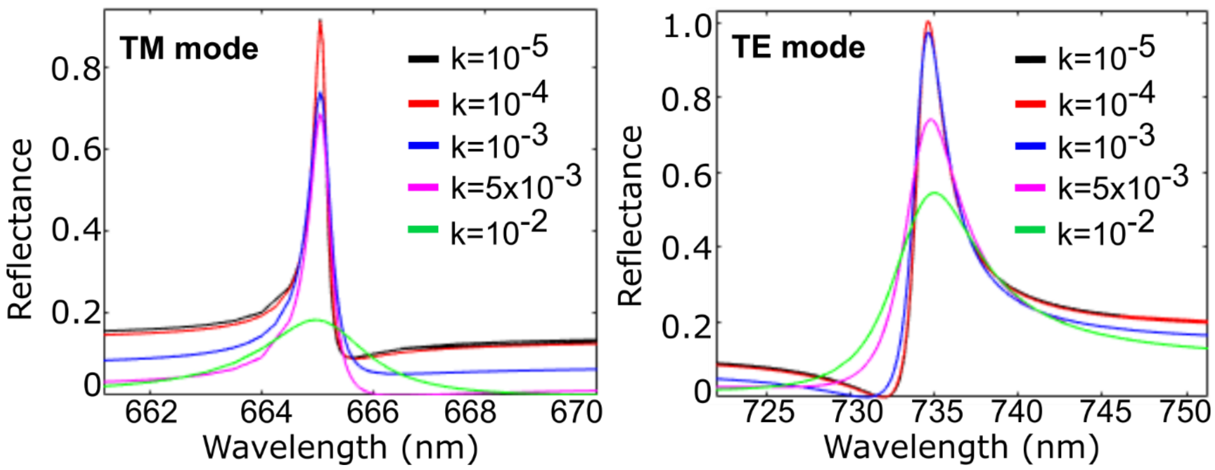

Supplementary Figure 2a. Reflection spectra of the nanohole array assuming different values of material loss for (left) the TM mode and (right) the TE mode.

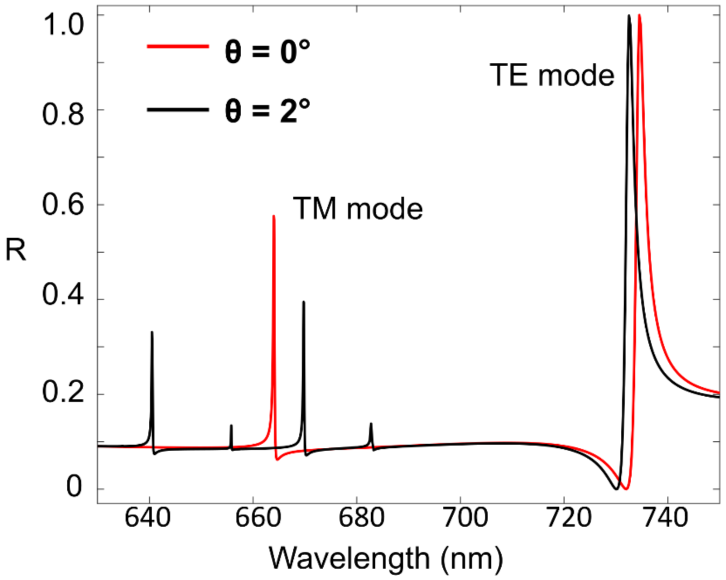

Supplementary Figure 2b. Reflectivity of the nanohole array for different angles of incidence.

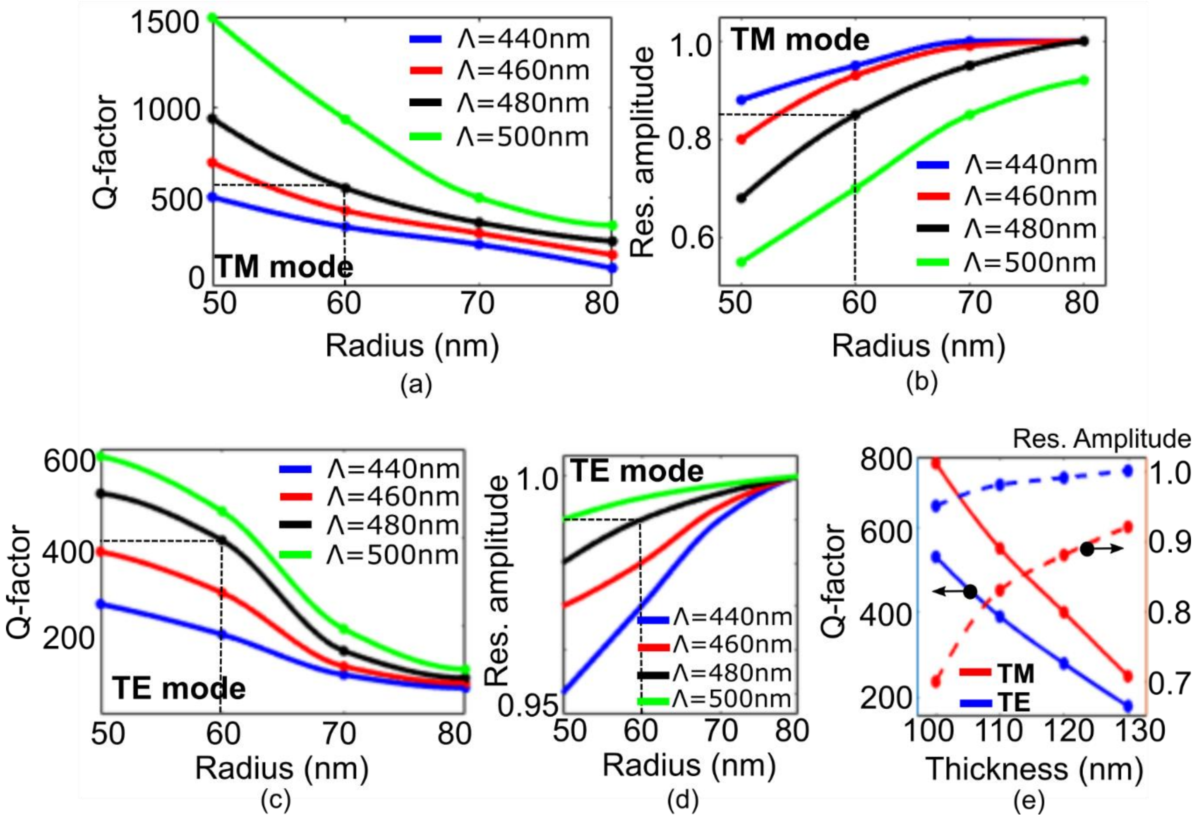

Supplementary Figure 3. Parametric analysis on the hole radius R and period Λ in the dielectric nanohole array. (a) Q-factor and (b) resonance amplitude vs. radius for different lattice periods for the TM mode. (c) Q-factor and (d) resonance amplitude vs. radius for the TE mode. The black dotted line represents the optimized configuration used in the experiments with R=60nm, t=110nm and Λ=480nm. (e) Q-factor and resonance amplitude vs. thickness of the dielectric slab for the TM mode and TE mode.

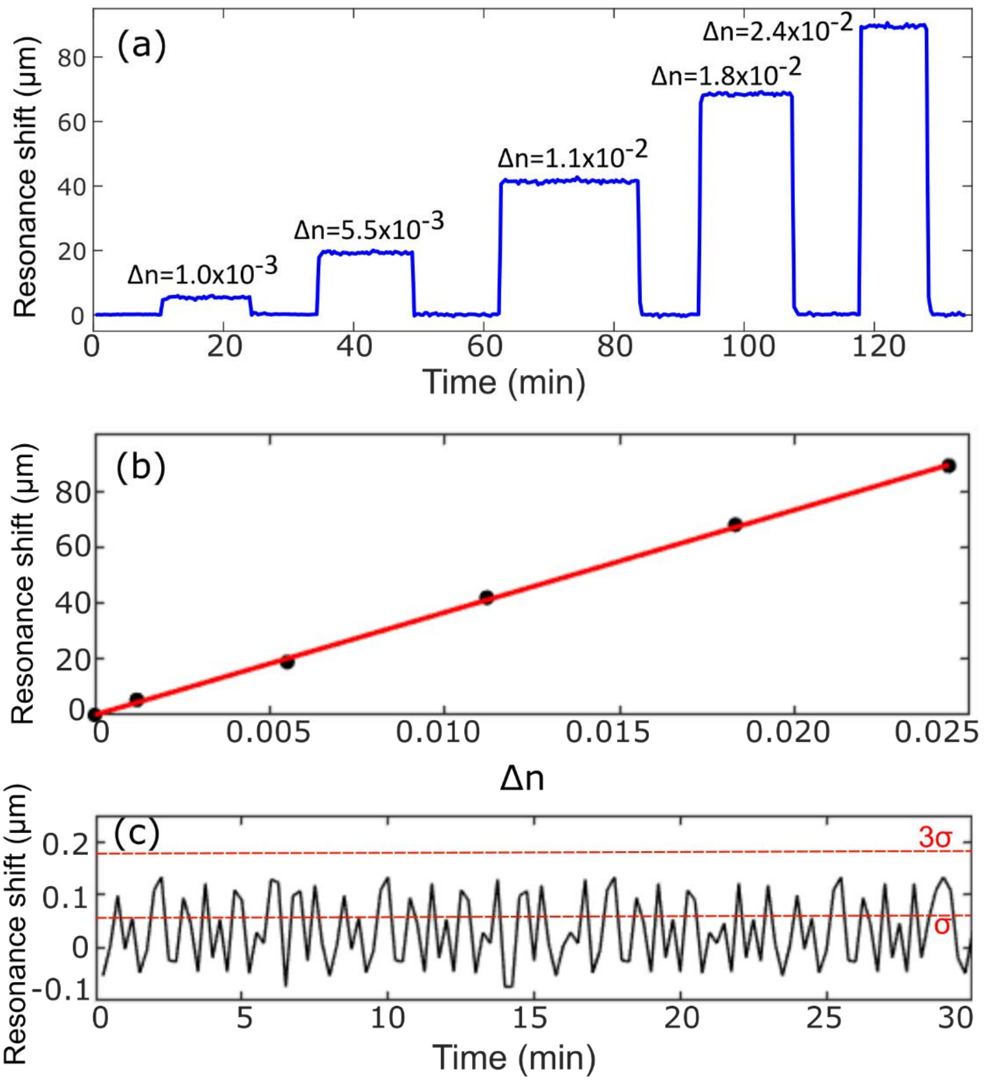

Supplementary Figure 5. (a) Resonance shift over time with different values of refractive index of the solution (b) Resonance shift (black dots) and corresponding linear fit (red curve) as a function of refractive index change of the solution. (c) Measurement of the noise limit in water for 30 min with $3\sigma = 0.183$ µm.

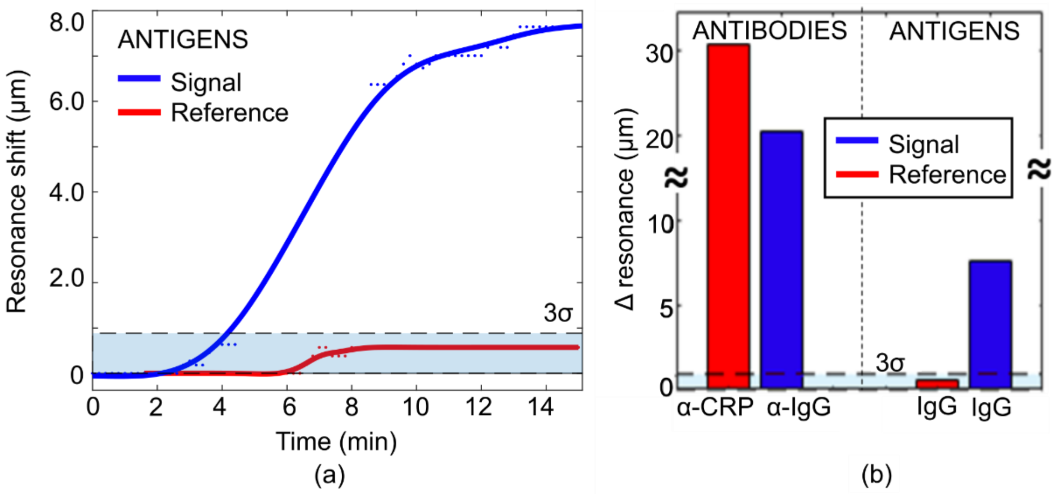

Supplementary Figure 6. *(a) Binding assay with 20 µg/mL IgG concentration in both signal (blue curve) and reference channel (red curve), which are functionalised with specific antibody anti-IgG and unspecific anti-CRP, respectively. (b) Histograms for the resonance shift observed during the assay for the antibodies binding and antigen binding in the signal and reference channel.*

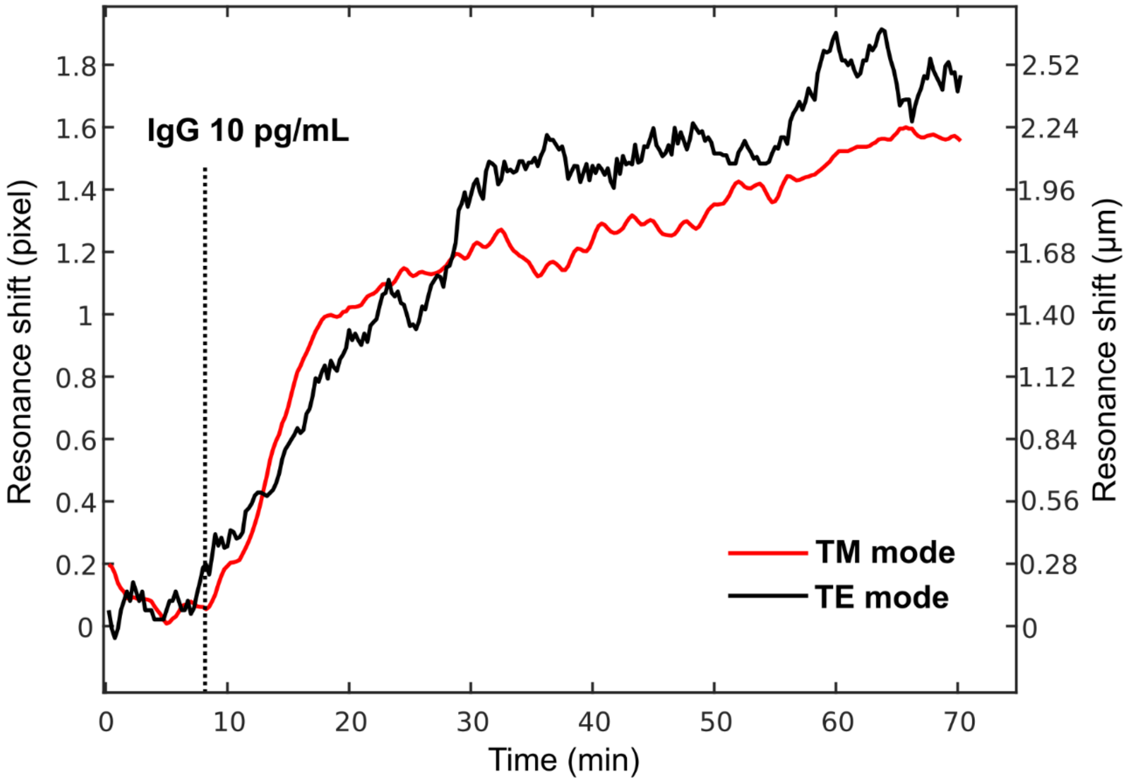

Supplementary Figure 7. Binding assay with chirped nanohole array for the TE (black curve) and TM (red curve) with 10 pg/mL of IgG.

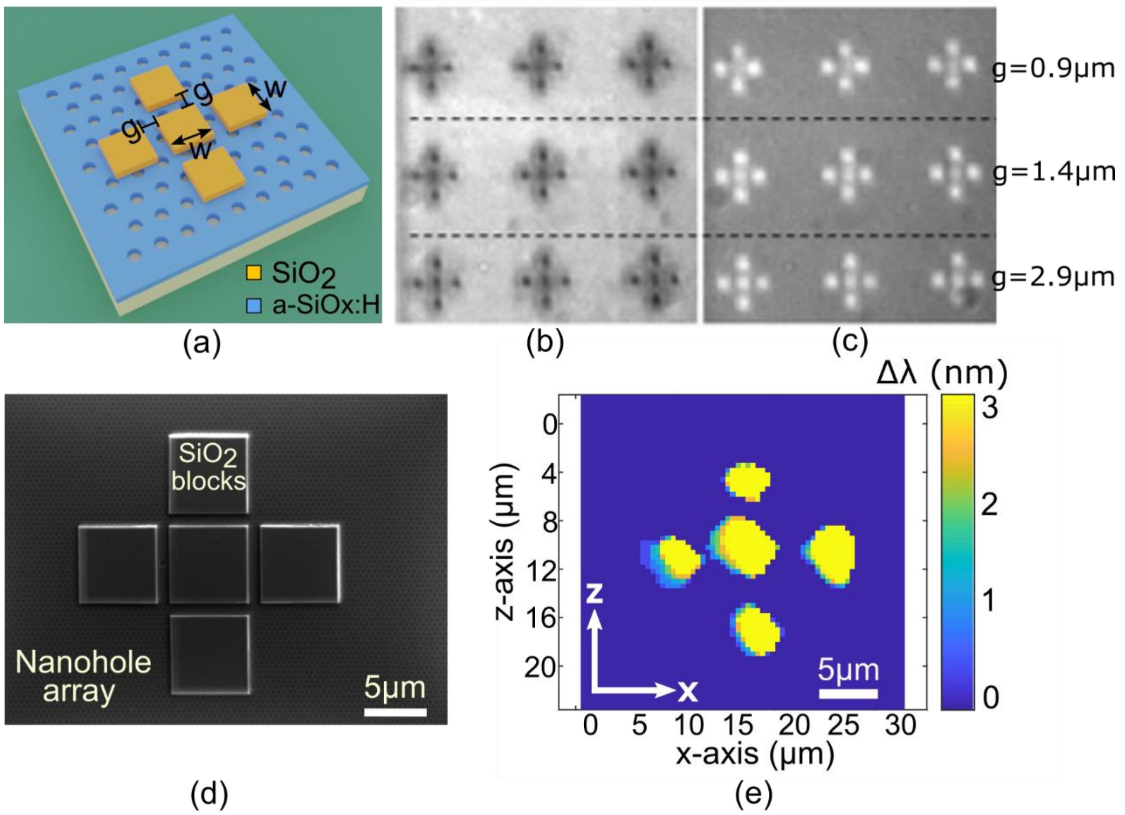

Supplementary Figure 8. (a) Schematic of SiO_2 blocks placed on top of the nanohole array with a width w = 5 μm and varying gap. (b,c) Images taken at the wavelength of the resonance peak of (b) the background and (c) the blocks. The separate blocks are clearly resolved, indicating a spatial resolution of better than 1μm; (d) SEM micrograph of the structure with blocks of gap g = 0.9 μm and (e) hyperspectral image of the same structure.

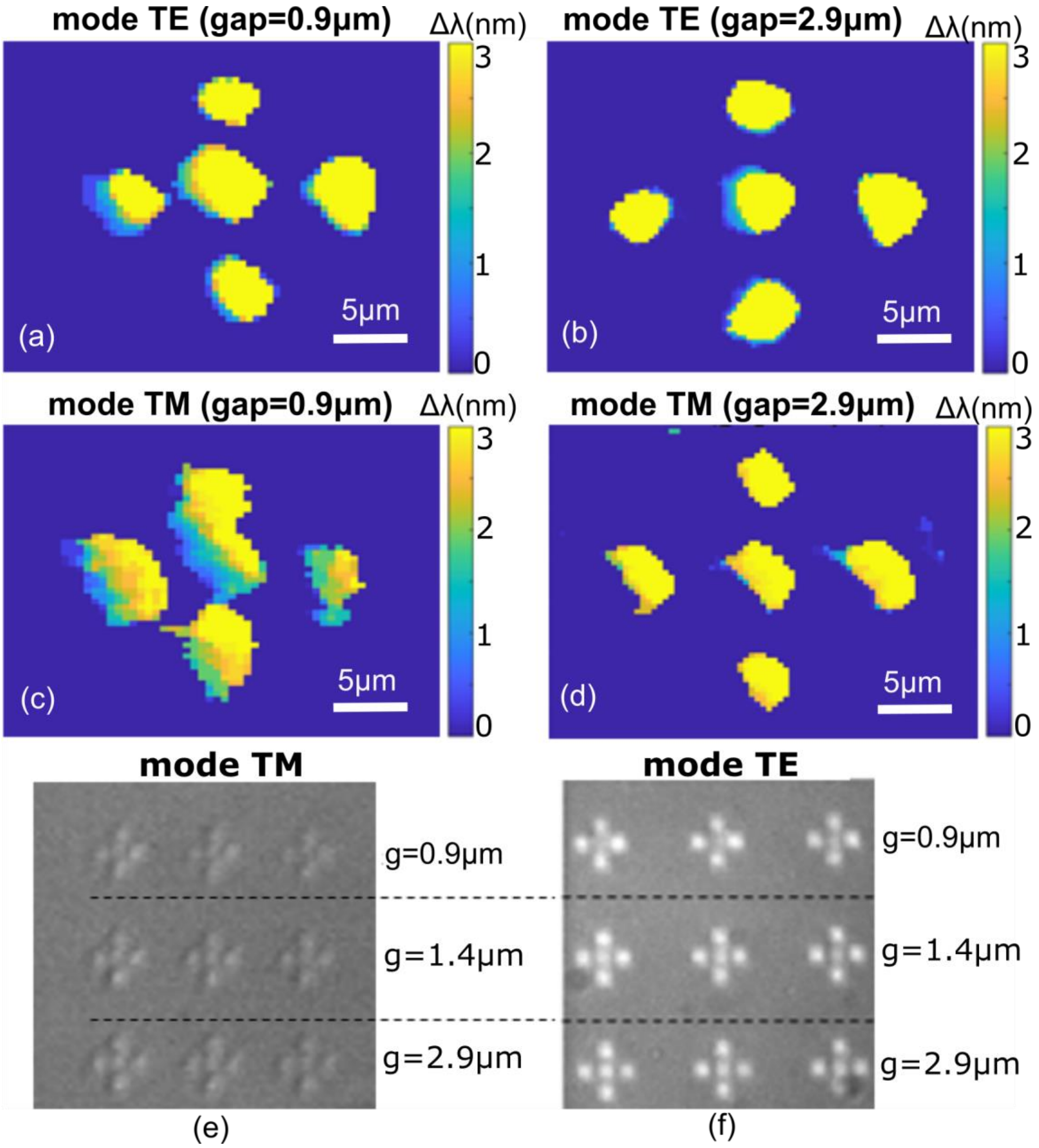

Supplementary Figure 9. Hyperspectral imaging of the blocks structure with the TE mode (a,b) and TM mode (c,d) with a blocks width of 5 µm and a gap size of 0.9 µm and 2.9 µm, respectively. Camera images taken at the resonance peak of the blocks for (e) the TM mode and (f) the TE mode.

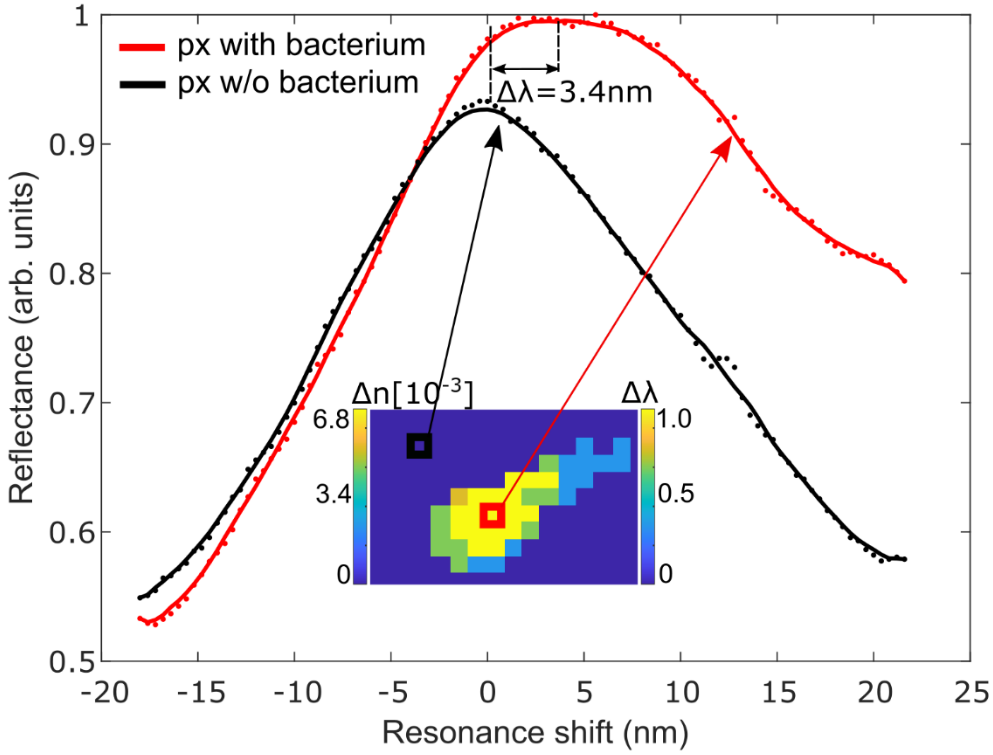

Supplementary Figure 10. Comparison of the resonance shift of two pixels (px) in the presence of a bacterium (red curve) and for the bare nanohole array without bacteria (black curve). The pixel size is 0.45 µm.

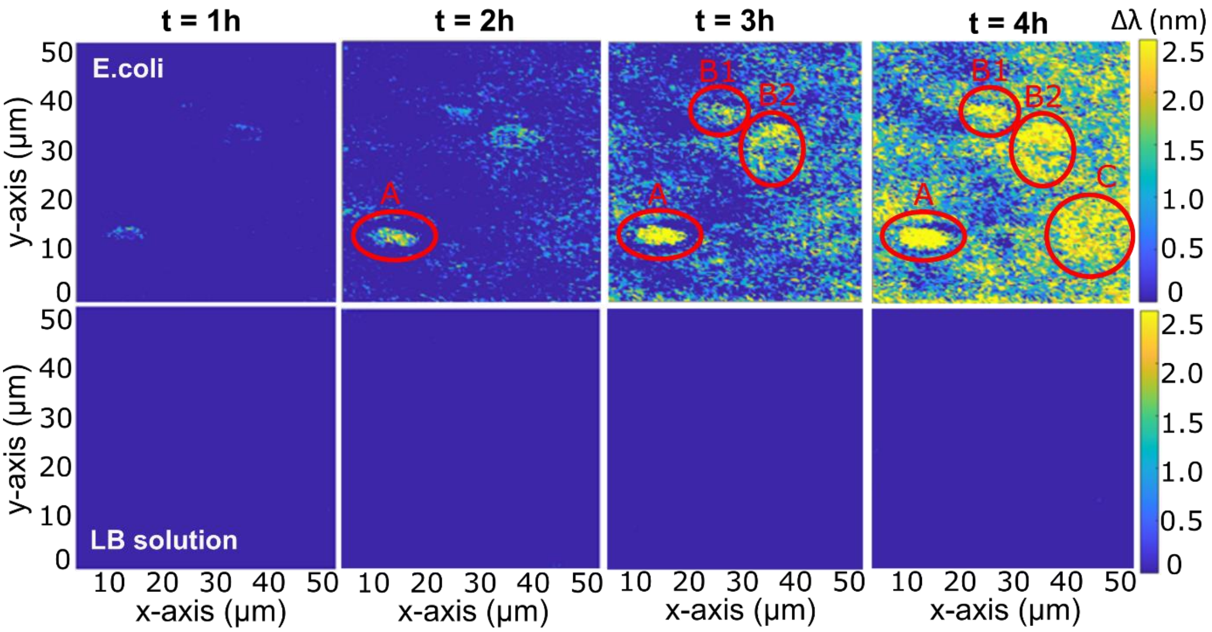

Supplementary Figure 11. Time dependence of the hyperspectral map of an area of 50 µm x 50 µm of the nanoholes array to monitor the growth of E.coli over time (top) compared to the control experiment with only LB medium without bacteria (bottom). A magnification of 20x is used to take each brightfield image.

Supplementary Tables

Supplementary Table 4. Comparison of the performance of dielectric nanoholes with the state-of-the-art of label free-biosensors with comparable structures

	$(R_{max}-R_{min})$	SNR_{res}	Q	Ss (nm/RIU)	FOM = SQ (nm/RIU)	LOD (fM)	LOD (pg/ml)
Plasmonic nanohole array [23]	0.2	/	40	30	$1.2x10^3$	$5x10^3$ [16]	145 [16]
1D GMR [28]<br>2D GMR [46]	0.8<br>0.8	/<br>/	240<br>130	31<br>/	$7.4x10^3$<br>/	$3x10^9$<br>$1.7x10^4$	$5x10^8$*<br>10^3
Dielectric metasurface [9]	0.6	~20	90	40	$3.6x10^3$	$8.5x10^4$	$4x10^4$
Dielectric metasurface [31]	0.2	~20	1300	53**	$6.9x10^4$	$6.6\ x10^7$ [39]	/
This work TE mode	0.8	160	300	20	$6x10^3$	~60	~10
This work TM mode	0.4	78	450	20	$9x10^3$	6.6	1

* The experiment has been conducted only with the reported concentration of analyte and/or the signal to noise ratio is still over the 3σ threshold, therefore the actual LOD could be potentially smaller than the reported value.

** This is a simulated value reported in [31].
